# Supplementary figures and images for: Global patterns of aegyptism without arbovirus
Source: PLoS Negl Trop Dis. 2021 May 5;15(5):e0009397. doi: 10.1371/journal.pntd.0009397 (PMC8128236; doi:10.1371/journal.pntd.0009397)

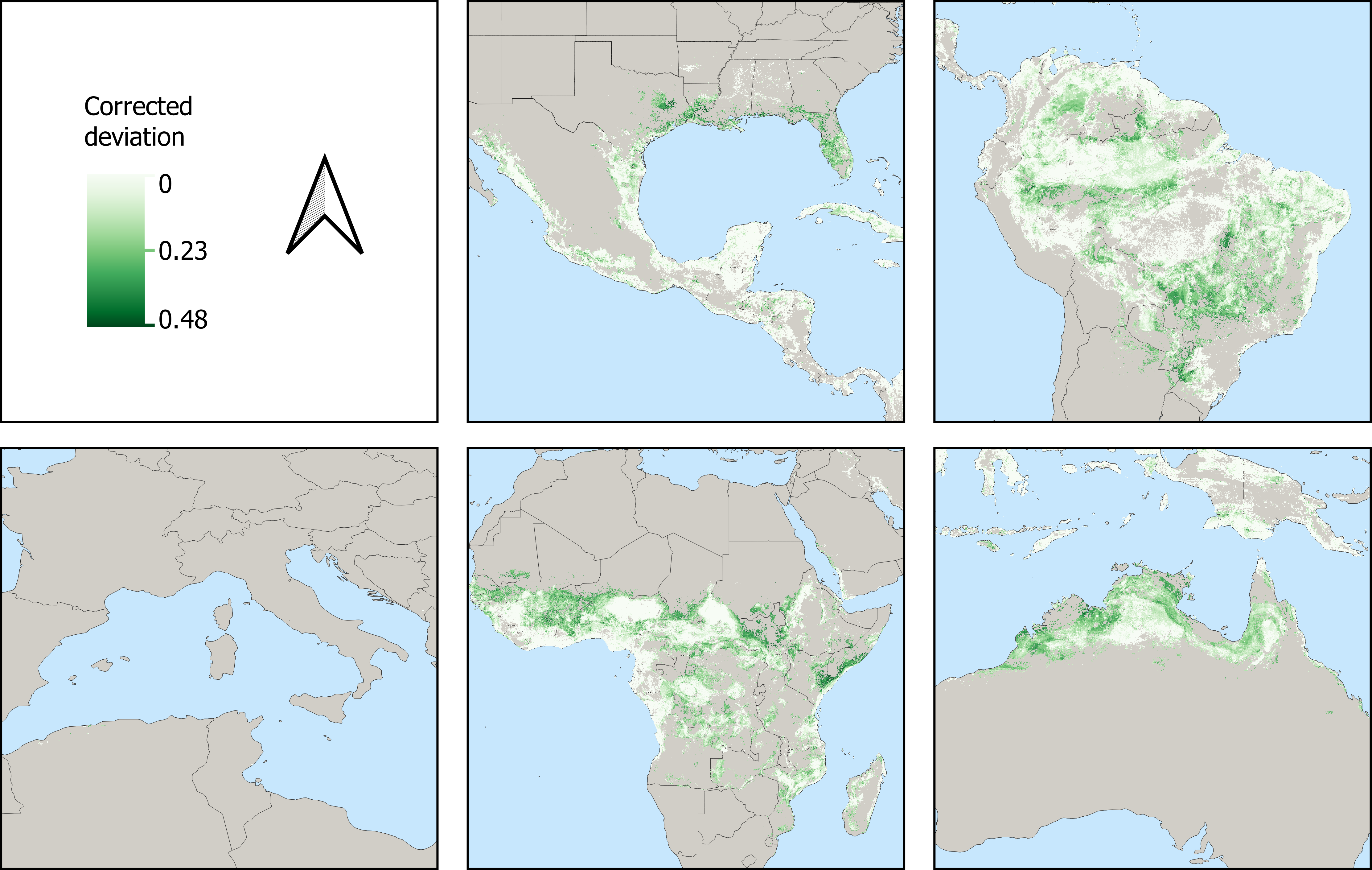

Supplement: S1 Fig — The map was created by the author using QGIS 3.10 (https://qgis.org/en/site/) with public domain map data from Natural Earth (https://www.naturalearthdata.com/downloads/50m-physical-vectors/) and U.S. Geological Survey (https://woodshole.er.usgs.gov/pubs/of2005-1071/data/background/us_bnds/state_boundsmeta.htm). (TIF) [file pntd.0009397.s001.tif]

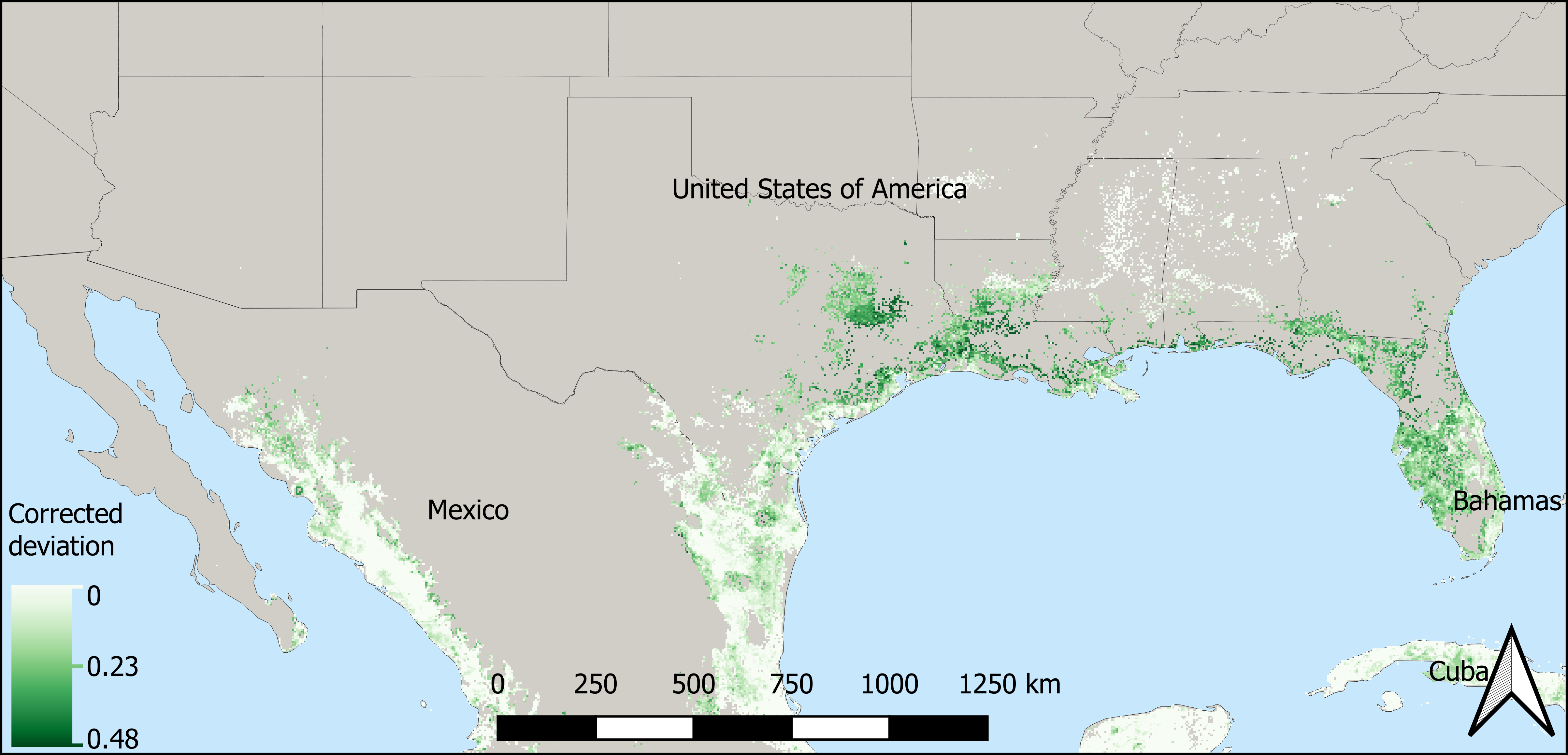

Supplement: S2 Fig — The map was created by the author using QGIS 3.10 (https://qgis.org/en/site/) with public domain map data from Natural Earth (https://www.naturalearthdata.com/downloads/50m-physical-vectors/) and U.S. Geological Survey (https://woodshole.er.usgs.gov/pubs/of2005-1071/data/background/us_bnds/state_boundsmeta.htm). (TIF) [file pntd.0009397.s002.tif]

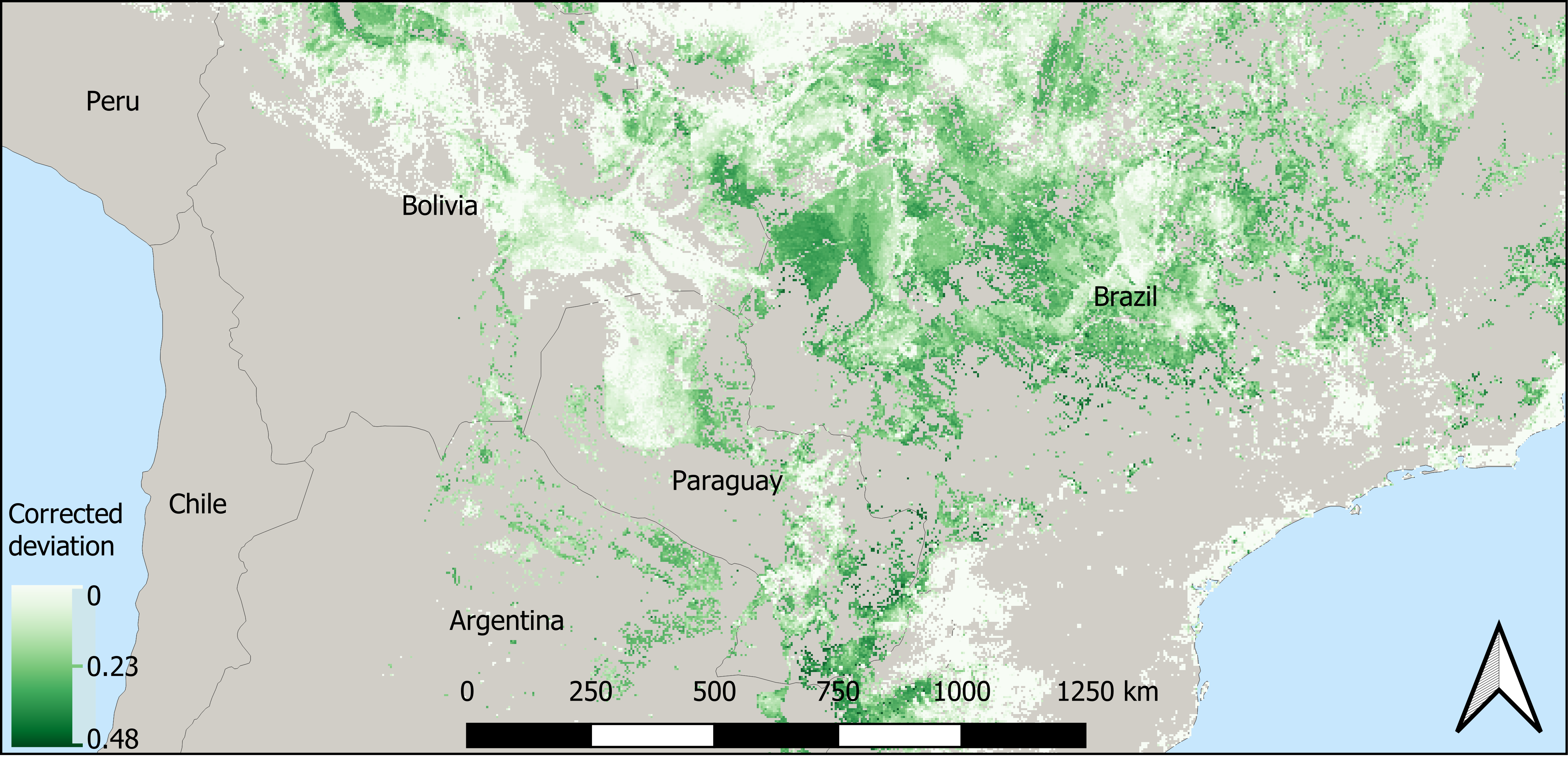

Supplement: S3 Fig — The map was created by the author using QGIS 3.10 (https://qgis.org/en/site/) with public domain map data from Natural Earth (https://www.naturalearthdata.com/downloads/50m-physical-vectors/) and U.S. Geological Survey (https://woodshole.er.usgs.gov/pubs/of2005-1071/data/background/us_bnds/state_boundsmeta.htm). (TIF) [file pntd.0009397.s003.tif]

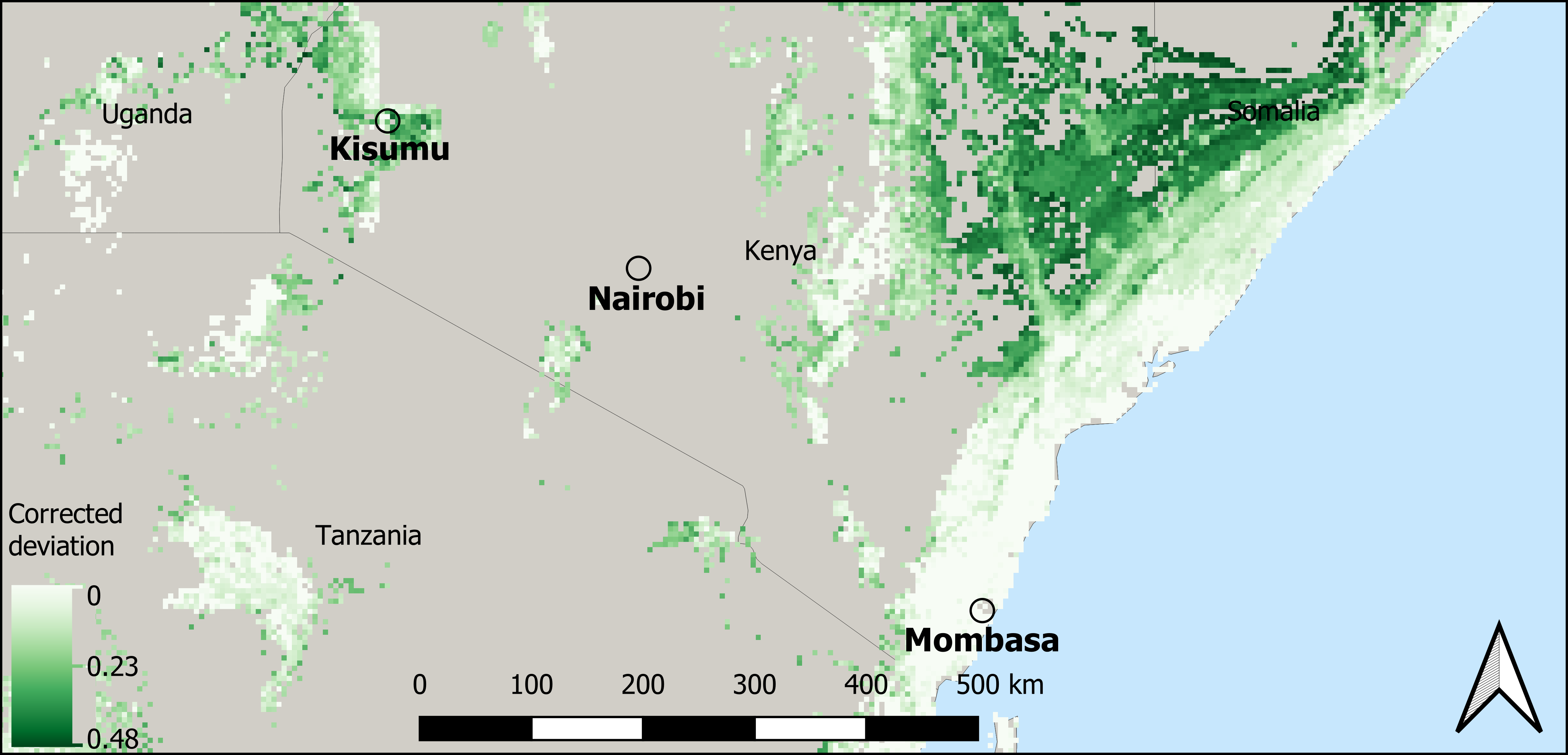

Supplement: S4 Fig — The map was created by the author using QGIS 3.10 (https://qgis.org/en/site/) with public domain map data from Natural Earth (https://www.naturalearthdata.com/downloads/50m-physical-vectors/) and U.S. Geological Survey (https://woodshole.er.usgs.gov/pubs/of2005-1071/data/background/us_bnds/state_boundsmeta.htm). (TIF) [file pntd.0009397.s004.tif]
