# Supplementary material for: Global patterns of aegyptism without arbovirus
Source: PLoS Negl Trop Dis. 2021 May 5;15(5):e0009397. doi: 10.1371/journal.pntd.0009397 (PMC8128236; doi:10.1371/journal.pntd.0009397)
Supplement: S1 Table — (DOCX) [file pntd.0009397.s005.docx]

**S1 Table.** Data sources for the global rasters used in this paper.

| **Variable** | **Scale** | **Data Source/URL** | **Reference** |
| --- | --- | --- | --- |
| Global distribution of *Ae. aegypti* | 5 x 5 km | https://datadryad.org/resource/doi:10.5061/dryad.47v3c | [1] |
| Global distribution of dengue | 5 x 5 km | https://figshare.com/s/d7d7871d00afe2870619 | [2] |
| Population Density | 1 x 1 km | <https://sedac.ciesin.columbia.edu/data/set/gpw-v4-population-density-rev11/data-download> | [3] |
| Gross domestic product | 1 x 1 km | <https://doi.org/10.5061/dryad.dk1j0> | [4] |
| Infant mortality rate | National/Subnational  1 x 1 km | <https://sedac.ciesin.columbia.edu/data/set/povmap-global-subnational-infant-mortality-rates-v2/data-download> | [5] |
| Temperature | 1 x 1 km | [https://www.worldclim.org/data/worldclim21.html#](https://www.worldclim.org/data/worldclim21.html) | [6] |
| Precipitation | 1 x 1 km | [https://www.worldclim.org/data/worldclim21.html#](https://www.worldclim.org/data/worldclim21.html) | [6] |
|  |  |  |  |

**References**

1. Kraemer, M.U.; Sinka, M.E.; Duda, K.A.; Mylne, A.Q.; Shearer, F.M.; Barker, C.M.; Moore, C.G.; Carvalho, R.G.; Coelho, G.E.; Van Bortel, W. The global distribution of the arbovirus vectors *Aedes aegypti* and *Ae. albopictus*. *elife* **2015**, *4*, e08347.

2. Messina, J.P.; Brady, O.J.; Golding, N.; Kraemer, M.U.; Wint, G.W.; Ray, S.E.; Pigott, D.M.; Shearer, F.M.; Johnson, K.; Earl, L. The current and future global distribution and population at risk of dengue. *Nature microbiology* **2019**, 1.

3. Center for International Earth Science Information Network - CIESIN - Columbia University. Gridded Population of the World, Version 4 (GPWv4): Population Density, Revision 11. NASA Socioeconomic Data and Applications Center (SEDAC): Palisades, NY, 2018.

4. Kummu, M.; Taka, M.; Guillaume, J.H. Gridded global datasets for gross domestic product and Human Development Index over 1990–2015. *Scientific data* **2018**, *5*, 180004.

5. Center for International Earth Science Information Network - CIESIN - Columbia University. Global Subnational Infant Mortality Rates, Version 2. NASA Socioeconomic Data and Applications Center (SEDAC): Palisades, NY, 2019.

6. Fick, S.E.; Hijmans, R.J. WorldClim 2: new 1‐km spatial resolution climate surfaces for global land areas. *International journal of climatology* **2017**, *37*, 4302-4315.
