# Supplementary material for: Global patterns of aegyptism without arbovirus
Source: PLoS Negl Trop Dis. 2021 May 5;15(5):e0009397. doi: 10.1371/journal.pntd.0009397 (PMC8128236; doi:10.1371/journal.pntd.0009397)
Supplement: S2 Table — White indicates the lower end of the spectrum, where Ae. aegypti occurrence and risk of dengue is nearly equal and high, and green represents the other end of the spectrum where Ae. aegypti can be found without dengue. Countries with 5 or fewer cells (5 km2) were removed from the table for brevity. (DOCX) [file pntd.0009397.s006.docx]

**S2 Table.** **Statistical summary of *Ae. aegypti* minus dengue deviation, by country.** White indicates the lower end of the spectrum, where *Ae. aegypti* occurrence and risk of dengue is nearly equal and high, and green represents the other end of the spectrum where *Ae.* *aegypti* can be found without dengue. Countries with 5 or fewer cells (5 km^2^) were removed from the table for brevity.

| **Country** | **Count** | **Mean Deviation** | **SD** | **Min** | **Max** | **Range** |
| --- | --- | --- | --- | --- | --- | --- |
| *Mauritania* | 1537 | 0.270822 | 0.076399 | 0 | 0.468075 | 0.468075 |
| *Niger* | 3212 | 0.241343 | 0.075063 | 0 | 0.43299 | 0.43299 |
| *Somalia* | 7144 | 0.210229 | 0.140471 | 0 | 0.476313 | 0.476313 |
| *Burkina Faso* | 9555 | 0.209692 | 0.08242 | 0 | 0.464294 | 0.464294 |
| *eSwatini* | 25 | 0.20527 | 0.030981 | 0.159627 | 0.267192 | 0.107565 |
| *S. Sudan* | 13965 | 0.189314 | 0.126751 | 0 | 0.461959 | 0.461959 |
| *Senegal* | 5230 | 0.182488 | 0.100429 | 0 | 0.449441 | 0.449441 |
| *South Africa* | 93 | 0.182341 | 0.080277 | 0 | 0.359835 | 0.359835 |
| *Botswana* | 15 | 0.182296 | 0.057185 | 0.084908 | 0.296197 | 0.211289 |
| *Kenya* | 8705 | 0.176906 | 0.130131 | 0 | 0.466411 | 0.466411 |
| *Argentina* | 3983 | 0.176614 | 0.105083 | 0 | 0.439078 | 0.439078 |
| *Netherlands* | 9 | 0.176019 | 0.089169 | 0 | 0.273538 | 0.273538 |
| *United States of America* | 12322 | 0.160234 | 0.127157 | 0 | 0.473787 | 0.473787 |
| *Mali* | 12187 | 0.143336 | 0.096475 | 0 | 0.444084 | 0.444084 |
| *Zimbabwe* | 272 | 0.140671 | 0.082867 | 0 | 0.417163 | 0.417163 |
| *Sudan* | 8478 | 0.133645 | 0.124151 | 0 | 0.44961 | 0.44961 |
| *Togo* | 2485 | 0.131907 | 0.111805 | 0 | 0.460853 | 0.460853 |
| *Paraguay* | 7689 | 0.131267 | 0.110785 | 0 | 0.459871 | 0.459871 |
| *Benin* | 5000 | 0.128714 | 0.099668 | 0 | 0.445118 | 0.445118 |
| *Australia* | 42383 | 0.125248 | 0.093226 | 0 | 0.449273 | 0.449273 |
| *Chad* | 12522 | 0.124446 | 0.122251 | 0 | 0.443918 | 0.443918 |
| *Gambia* | 423 | 0.121773 | 0.076891 | 0 | 0.375483 | 0.375483 |
| *Algeria* | 33 | 0.120733 | 0.114471 | 0 | 0.311777 | 0.311777 |
| *Zambia* | 7392 | 0.119709 | 0.0742 | 0 | 0.319709 | 0.319709 |
| *Ghana* | 9599 | 0.115504 | 0.114871 | 0 | 0.455999 | 0.455999 |
| *Singapore* | 21 | 0.114384 | 0.064616 | 0 | 0.214392 | 0.214392 |
| *Namibia* | 351 | 0.110637 | 0.074253 | 0 | 0.284943 | 0.284943 |
| *Mauritius* | 84 | 0.109631 | 0.099659 | 0 | 0.430635 | 0.430635 |
| *Dem. Rep. Congo* | 49377 | 0.102295 | 0.093354 | 0 | 0.441369 | 0.441369 |
| *Saudi Arabia* | 1314 | 0.098834 | 0.100066 | 0 | 0.42865 | 0.42865 |
| *Guinea-Bissau* | 1155 | 0.098167 | 0.08264 | 0 | 0.423113 | 0.423113 |
| *Mozambique* | 18281 | 0.095912 | 0.088501 | 0 | 0.441035 | 0.441035 |
| *Afghanistan* | 29 | 0.093353 | 0.105213 | 0 | 0.322509 | 0.322509 |
| *Uganda* | 4281 | 0.092651 | 0.087806 | 0 | 0.43509 | 0.43509 |
| *Djibouti* | 585 | 0.091013 | 0.092261 | 0 | 0.380496 | 0.380496 |
|  | | | | | | |
| *Ethiopia* | 14335 | 0.089882 | 0.103374 | 0 | 0.473594 | 0.473594 |
| *Turkey* | 125 | 0.086916 | 0.103747 | 0 | 0.347339 | 0.347339 |
| *Tanzania* | 12375 | 0.086016 | 0.086437 | 0 | 0.401491 | 0.401491 |
| *Brazil* | 251421 | 0.080467 | 0.09552 | 0 | 0.454705 | 0.454705 |
| *Libya* | 10 | 0.076496 | 0.074334 | 0 | 0.208514 | 0.208514 |
| *Angola* | 13627 | 0.075843 | 0.081154 | 0 | 0.322925 | 0.322925 |
| *Central African Rep.* | 23993 | 0.075756 | 0.086161 | 0 | 0.41307 | 0.41307 |
| *Côte d'Ivoire* | 13634 | 0.075138 | 0.101408 | 0 | 0.46961 | 0.46961 |
| *Congo* | 8203 | 0.074303 | 0.080669 | 0 | 0.437049 | 0.437049 |
| *Bolivia* | 20245 | 0.072595 | 0.086744 | 0 | 0.420899 | 0.420899 |
| *Bangladesh* | 6589 | 0.071522 | 0.080114 | 0 | 0.391463 | 0.391463 |
| *Madagascar* | 10491 | 0.070617 | 0.078189 | 0 | 0.420165 | 0.420165 |
| *Spain* | 7 | 0.068162 | 0.086906 | 0 | 0.252066 | 0.252066 |
| *Malawi* | 1112 | 0.067902 | 0.086921 | 0 | 0.360916 | 0.360916 |
| *Guam* | 12 | 0.066335 | 0.060257 | 0 | 0.163893 | 0.163893 |
| *Israel* | 25 | 0.064371 | 0.102963 | 0 | 0.332563 | 0.332563 |
| *Cameroon* | 12896 | 0.06422 | 0.085394 | 0 | 0.421467 | 0.421467 |
| *Hong Kong* | 29 | 0.062199 | 0.077636 | 0 | 0.302333 | 0.302333 |
| *Colombia* | 36374 | 0.061137 | 0.075168 | 0 | 0.424601 | 0.424601 |
| *China* | 25740 | 0.060055 | 0.078603 | 0 | 0.43586 | 0.43586 |
| *Nigeria* | 40458 | 0.058021 | 0.079571 | 0 | 0.443585 | 0.443585 |
| *Uruguay* | 21 | 0.056554 | 0.063163 | 0 | 0.171148 | 0.171148 |
| *Eritrea* | 1507 | 0.056547 | 0.075275 | 0 | 0.437792 | 0.437792 |
| *Aruba* | 6 | 0.056413 | 0.042776 | 0 | 0.140137 | 0.140137 |
| *Haiti* | 1125 | 0.055947 | 0.081108 | 0 | 0.407427 | 0.407427 |
| *Peru* | 28758 | 0.053313 | 0.0784 | 0 | 0.448863 | 0.448863 |
| *Myanmar* | 18541 | 0.050836 | 0.072837 | 0 | 0.431414 | 0.431414 |
| *Bhutan* | 469 | 0.049784 | 0.067289 | 0 | 0.275594 | 0.275594 |
| *Venezuela* | 28011 | 0.048846 | 0.066544 | 0 | 0.426449 | 0.426449 |
| *Brunei* | 254 | 0.047027 | 0.05883 | 0 | 0.31676 | 0.31676 |
| *India* | 131543 | 0.046476 | 0.067103 | 0 | 0.482278 | 0.482278 |
| *Guinea* | 10106 | 0.04542 | 0.071513 | 0 | 0.463709 | 0.463709 |
| *Guyana* | 7224 | 0.044463 | 0.070662 | 0 | 0.408294 | 0.408294 |
| *Nepal* | 2929 | 0.043455 | 0.066457 | 0 | 0.421316 | 0.421316 |
| *Puerto Rico* | 371 | 0.041801 | 0.057873 | 0 | 0.288344 | 0.288344 |
| *Burundi* | 67 | 0.040727 | 0.061973 | 0 | 0.255066 | 0.255066 |
| *Honduras* | 3574 | 0.038958 | 0.071488 | 0 | 0.374007 | 0.374007 |
| *Japan* | 798 | 0.037542 | 0.069031 | 0 | 0.45001 | 0.45001 |
| *Taiwan* | 1023 | 0.036766 | 0.05761 | 0 | 0.458133 | 0.458133 |
| *Mexico* | 30091 | 0.036353 | 0.062812 | 0 | 0.442393 | 0.442393 |
| *Liberia* | 2271 | 0.03601 | 0.063775 | 0 | 0.353039 | 0.353039 |
| *Greece* | 38 | 0.035851 | 0.083678 | 0 | 0.328919 | 0.328919 |
| *Cuba* | 5161 | 0.03374 | 0.054247 | 0 | 0.357021 | 0.357021 |
| *Pakistan* | 11821 | 0.033113 | 0.0626 | 0 | 0.435065 | 0.435065 |
| *Cambodia* | 6908 | 0.032395 | 0.057148 | 0 | 0.394668 | 0.394668 |
| *Sierra Leone* | 2137 | 0.032228 | 0.050699 | 0 | 0.309454 | 0.309454 |
| *Suriname* | 3890 | 0.028387 | 0.04457 | 0 | 0.309013 | 0.309013 |
| *Dominican Rep.* | 1649 | 0.027419 | 0.053722 | 0 | 0.341377 | 0.341377 |
| *New Caledonia* | 856 | 0.026595 | 0.045528 | 0 | 0.27335 | 0.27335 |
| *Tonga* | 17 | 0.02625 | 0.043202 | 0 | 0.131397 | 0.131397 |
| *Curaçao* | 20 | 0.023596 | 0.036404 | 0 | 0.111911 | 0.111911 |
| *Barbados* | 18 | 0.023528 | 0.032648 | 0 | 0.102893 | 0.102893 |
| *Thailand* | 20176 | 0.022727 | 0.044623 | 0 | 0.416792 | 0.416792 |
| *Syria* | 10 | 0.02266 | 0.037387 | 0 | 0.109027 | 0.109027 |
| *Yemen* | 1884 | 0.020873 | 0.049575 | 0 | 0.345343 | 0.345343 |
| *Papua New Guinea* | 9466 | 0.020219 | 0.047637 | 0 | 0.414984 | 0.414984 |
| *Gabon* | 7059 | 0.020012 | 0.046014 | 0 | 0.277781 | 0.277781 |
| *Belize* | 817 | 0.019791 | 0.043049 | 0 | 0.295161 | 0.295161 |
| *Laos* | 3776 | 0.018606 | 0.04681 | 0 | 0.302114 | 0.302114 |
| *Turks and Caicos Is.* | 11 | 0.017997 | 0.039329 | 0 | 0.134937 | 0.134937 |
| *Comoros* | 51 | 0.017759 | 0.039172 | 0 | 0.169279 | 0.169279 |
| *Indonesia* | 61849 | 0.017626 | 0.044067 | 0 | 0.458635 | 0.458635 |
| *Philippines* | 8957 | 0.017602 | 0.036593 | 0 | 0.339598 | 0.339598 |
| *Nicaragua* | 2649 | 0.017339 | 0.045104 | 0 | 0.385047 | 0.385047 |
| *Fr. Polynesia* | 41 | 0.017271 | 0.050807 | 0 | 0.27058 | 0.27058 |
| *Cabo Verde* | 86 | 0.016377 | 0.035636 | 0 | 0.155874 | 0.155874 |
| *Antigua and Barb.* | 7 | 0.016027 | 0.0392 | 0 | 0.112023 | 0.112023 |
| *St. Vin. and Gren.* | 13 | 0.016003 | 0.028461 | 0 | 0.100453 | 0.100453 |
| *Sri Lanka* | 2757 | 0.015825 | 0.034071 | 0 | 0.325308 | 0.325308 |
| *Guatemala* | 2846 | 0.014808 | 0.040273 | 0 | 0.345901 | 0.345901 |
| *Iraq* | 1334 | 0.013824 | 0.040605 | 0 | 0.313613 | 0.313613 |
| *U.S. Virgin Is.* | 9 | 0.013004 | 0.024272 | 0 | 0.067725 | 0.067725 |
| *Ecuador* | 4006 | 0.012696 | 0.040101 | 0 | 0.320929 | 0.320929 |
| *El Salvador* | 661 | 0.011557 | 0.0362 | 0 | 0.251234 | 0.251234 |
| *Vietnam* | 11576 | 0.010152 | 0.029572 | 0 | 0.325532 | 0.325532 |
| *Cayman Is.* | 6 | 0.010046 | 0.021944 | 0 | 0.058882 | 0.058882 |
| *Jordan* | 16 | 0.009814 | 0.020189 | 0 | 0.079458 | 0.079458 |
| *Malaysia* | 9712 | 0.009607 | 0.028345 | 0 | 0.352326 | 0.352326 |
| *Dominica* | 26 | 0.008615 | 0.0257 | 0 | 0.10747 | 0.10747 |
| *Jamaica* | 450 | 0.008046 | 0.030807 | 0 | 0.262963 | 0.262963 |
| *Trinidad and Tobago* | 213 | 0.0052 | 0.015503 | 0 | 0.09955 | 0.09955 |
| *South Korea* | 23 | 0.004973 | 0.019298 | 0 | 0.093969 | 0.093969 |
| *Timor-Leste* | 536 | 0.004884 | 0.016336 | 0 | 0.112183 | 0.112183 |
| *Costa Rica* | 692 | 0.004871 | 0.023694 | 0 | 0.213336 | 0.213336 |
| *United Arab Emirates* | 303 | 0.004546 | 0.021288 | 0 | 0.140047 | 0.140047 |
| *Saint Lucia* | 28 | 0.004106 | 0.012973 | 0 | 0.055122 | 0.055122 |
| *Panama* | 1704 | 0.004076 | 0.019316 | 0 | 0.216352 | 0.216352 |
| *Eq. Guinea* | 955 | 0.003257 | 0.017095 | 0 | 0.19235 | 0.19235 |
| *Iran* | 2937 | 0.003238 | 0.020055 | 0 | 0.313523 | 0.313523 |
| *Bahamas* | 425 | 0.002046 | 0.010936 | 0 | 0.104678 | 0.104678 |
| *Vanuatu* | 325 | 0.001953 | 0.014117 | 0 | 0.185129 | 0.185129 |
| *Oman* | 1695 | 0.001612 | 0.012964 | 0 | 0.183969 | 0.183969 |
| *Grenada* | 15 | 0.001142 | 0.002923 | 0 | 0.009292 | 0.009292 |
| *Solomon Is.* | 140 | 0.000664 | 0.005397 | 0 | 0.051616 | 0.051616 |
| *Fiji* | 434 | 0.000282 | 0.002888 | 0 | 0.039222 | 0.039222 |
| *Chile* | 6 | 0 | 0 | 0 | 0 | 0 |
| *Bahrain* | 15 | 0 | 0 | 0 | 0 | 0 |
| *Egypt* | 24 | 0 | 0 | 0 | 0 | 0 |
| *Georgia* | 22 | 0 | 0 | 0 | 0 | 0 |
| *Rwanda* | 7 | 0 | 0 | 0 | 0 | 0 |
| *Montenegro* | 6 | 0 | 0 | 0 | 0 | 0 |
| *Samoa* | 34 | 0 | 0 | 0 | 0 | 0 |
| *São Tomé and Principe* | 30 | 0 | 0 | 0 | 0 | 0 |
| *Niue* | 7 | 0 | 0 | 0 | 0 | 0 |
| *N. Mariana Is.* | 14 | 0 | 0 | 0 | 0 | 0 |
