# Supplementary material for: Global patterns of aegyptism without arbovirus
Source: PLoS Negl Trop Dis. 2021 May 5;15(5):e0009397. doi: 10.1371/journal.pntd.0009397 (PMC8128236; doi:10.1371/journal.pntd.0009397)
Supplement: Attachment — Submitted filename: Response to Reviewers.docx [file pntd.0009397.s007.docx]

**Global patterns of aegyptism without arbovirus: Response to Reviewers**

**Key Review Criteria Required for Acceptance**

As you describe the new analyses required for acceptance, please consider the following:

**Methods**

-Are the objectives of the study clearly articulated with a clear testable hypothesis stated?

-Is the study design appropriate to address the stated objectives?

-Is the population clearly described and appropriate for the hypothesis being tested?

-Is the sample size sufficient to ensure adequate power to address the hypothesis being tested?

-Were correct statistical analysis used to support conclusions?

-Are there concerns about ethical or regulatory requirements being met?

Reviewer #1: See my overall review in the general comments

Reviewer #2: Methods are appropriate.

--------------------

**Results**

-Does the analysis presented match the analysis plan?

-Are the results clearly and completely presented?

-Are the figures (Tables, Images) of sufficient quality for clarity?

Reviewer #1: See my overall review in the general comments

Reviewer #2: Results are clear.

--------------------

**Conclusions**

-Are the conclusions supported by the data presented?

-Are the limitations of analysis clearly described?

-Do the authors discuss how these data can be helpful to advance our understanding of the topic under study?

-Is public health relevance addressed?

Reviewer #1: See my overall review in the general comments

Reviewer #2: Yes.

--------------------

**Editorial and Data Presentation Modifications**

Use this section for editorial suggestions as well as relatively minor modifications of existing data that would enhance clarity. If the only modifications needed are minor and/or editorial, you may wish to recommend “Minor Revision” or “Accept”.

Reviewer #1: See my overall review in the general comments

Reviewer #2: Minor Revision.

--------------------

**Summary and General Comments**

Use this section to provide overall comments, discuss strengths/weaknesses of the study, novelty, significance, general execution and scholarship. You may also include additional comments for the author, including concerns about dual publication, research ethics, or publication ethics. If requesting major revision, please articulate the new experiments that are needed.

Reviewer #1: This paper describes a modeling analysis of the differential between projected Ae aegypti populations and a model of the likelihood of dengue transmission in an attempt to investgate why some areas with the vector lack dengue. This is an important topic as the primary vector of dengue, Ae aegypti, is spreading to new areas. In particular, parts of the southwestern United States including urban areas in California and Arizona, have recently been infested with this mosquito. And it appears as though the populations are well established in those areas. In addition, dengue is noticeable by its absence in these areas.

The issue I have with this work is that it really is based on modeling projections that were conducted five to six years ago. Indeed the models were based on input up to 2015, and so are somewhat dated. I was hoping that the paper would include a benchtop exercise / literature review of the known and current areas with both vector and dengue transmission. The Aedes model makes some embarrassing whiffs, showing Ae. aegypti widespread across northern Australia (it is limited to coastal northern Queensland), and missing the populations in the southwestern United States. The dengue model is likewise based on projections, with dengue probable in much of the SE USA despite a paucity of transmission there.

What are we to make of a study that is the product of 2 less than perfect models? Is it error squared? It certainly does not reflect the title of “Global patterns…” that indicate real distributional data. So I think that you need to describe this work as a modeling exercise, an explorative dive into potential links between vector, virus and the environment.

Author response: We agree that this study is an exploratory start into these concepts using previously published global distribution maps. As you point out, these previous maps used disjunct empirical data on *Ae. aegypti* or human dengue virus cases to model and then predict global habitat suitability are not perfect. While good as a global perspective, we also now point out in the manuscript that these maps are predictions of suitability which do not necessarily reflect occurrence. The authors intend to update these global predictions in the future but these are large studies which are outside the scope of this current manuscript. Also, the year of 2015 is good given that we had both the Ae. aegypti and dengue models as well as environmental, meteorological, and demographic data all for this similar time period. We made changes to the manuscript to emphasize that this study builds on the previous models of habitat suitability for 2015. We hope that this study provides a framework for future studies to improve the precision of the analysis.

I also feel that the paper needs more thorough discussion of reasons why dengue may not occur in areas where Ae. aegypti does. This would be a function of, as you did point out, the development and wealth of the population such that they have access to piped water, window screening and air conditioning. This of course is brought out in the Reiter 2003 publication that you did cite but perhaps a brief discussion of the role that Ae. aegypti as an indoor mosquito has in urban dengue transmission. Other aspects include herd immunity, access to endemic cases, insecticide resistance. Other aspects that you should discuss is that dengue occurs in areas where there are no Ae. aegypti, see outbreaks in southeast China and Japan that have been vectored by Ae. albopictus.

Author response: Thank you for this recommendation. We have expanded our discussion to include considerations as you suggested. (Please see lines 278-293 of the revised manuscript.)

A few other suggestions:

1. The colours/shades on the map do not work well. I have a very hard time seeing the pale yellow. I suggest seeking the following website: https://colorbrewer2.org and revising.

Author response: Thanks for your suggested changes to the map colors. We revised the colors on the figure to improve the ability to interpret the results.

2. Please state what sort of populations, mosquito or human, when used.

Author response: We added the word ‘human’ in front of most instances referring to human population in the manuscript (i.e. lines 28, 29, 45, 131, 134, 153, etc.). Other instances of ‘population’ are already qualified with the species name *Ae. aegypti* (i.e. lines 15, 30, 65, 66, etc.)

Reviewer #2: Olson et al. present results on development of a global map detailing probability estimates of Aedes aegypti occurrence and likelihood for dengue transmission. Overall, the manuscript is well-written and presents results that should be of interest to local/regional health authorities as they look to control arbovirus transmission. I only have some minor comments for the authors to consider:

Lines 78-81: these lines highlight a general pattern in the manuscript to alternate between the use of “dengue” and “DEN” – I’d suggest picking one and being consistent.

Author response: Thank you for this suggestion. We have changed all references to “dengue” for clarity and consistency in the manuscript.

Lines 138-143: it seems important to justify up front why the choice was made to combine monthly information on temperature and precipitation into annual mean temperatures and cumulative rainfall. This seems to be an overly simplistic approach that ignores the known effects on basic biology and population dynamics, e.g., maximum/minimum temperatures and timing and amounts of rainfall. As the authors indicate (lines 255-264) that choice likely impacted the predictability of their results.

Author response: We acknowledge that cumulative precipitation and average annual rainfall reduces the granularity that would perhaps reveal more associations and increase the fidelity of our predictions. However, we chose to limit the scope of this modelling study to provide a foundation for future studies which could explore environmental influences on *Aedes aegypti* infestation and dengue transmission.

--------------------

PLOS authors have the option to publish the peer review history of their article (what does this mean? If published, this will include your full peer review and any attached files.

If you choose “no”, your identity will remain anonymous but your review may still be made public.

Do you want your identity to be public for this peer review? For information about this choice, including consent withdrawal, please see our Privacy Policy

Reviewer #1: No

Reviewer #2: No

Figure Files:

While revising your submission, please upload your figure files to the Preflight Analysis and Conversion Engine (PACE) digital diagnostic tool, PACE helps ensure that figures meet PLOS requirements. To use PACE, you must first register as a user. Then, login and navigate to the UPLOAD tab, where you will find detailed instructions on how to use the tool. If you encounter any issues or have any questions when using PACE, please email us at [figures@plos.org](mailto:figures@plos.org).

Data Requirements:

Please note that, as a condition of publication, PLOS' data policy requires that you make available all data used to draw the conclusions outlined in your manuscript. Data must be deposited in an appropriate repository, included within the body of the manuscript, or uploaded as supporting information. This includes all numerical values that were used to generate graphs, histograms etc.. For an example see here:

Reproducibility:

To enhance the reproducibility of your results, PLOS recommends that you deposit laboratory protocols in protocols.io, where a protocol can be assigned its own identifier (DOI) such that it can be cited independently in the future. For instructions see https://journals.plos.org/plosntds/s/submission-guidelines

__________________________________________________

In compliance with data protection regulations, you may request that we remove your personal registration details at any time. Please contact the publication office if you have any questions.
